# Is there a prognostic value of tumor location among Chinese patients with colorectal cancer?

**DOI:** 10.18632/oncotarget.16305

**Published:** 2017-03-17

**Authors:** Fangqi Liu, Cong Li, Huixun Jia, Li Yang, Yuchen Wu, Jiang Zhao, Sanjun Cai, Ji Zhu, Ye Xu

**Affiliations:** ^1^ Department of Colorectal Surgery, Fudan University Shanghai Cancer Center, Shanghai 200032, China, Department of Oncology, Shanghai Medical College, Fudan University, Shanghai 200032, China; ^2^ Department of Clinical Statistics, Fudan University Shanghai Cancer Center, Shanghai 200032, China, Department of Oncology, Shanghai Medical College, Fudan University, Shanghai 200032, China; ^3^ Department of Radiation Oncology, Fudan University Shanghai Cancer Center, Shanghai 200032, China, Department of Oncology, Shanghai Medical College, Fudan University, Shanghai 200032, China

**Keywords:** colorectal cancer, tumor location, prognosis, left-sided colon cancer, right-sided colon cancer

## Abstract

Differences in epidemiology, pathological features, and molecular pathogeneses have been observed according to primary tumor location in colorectal cancer (CRC). However, predicting CRC survival by tumor location remains controversial. Therefore, we compared the pathological characteristics, molecular features, and prognoses of right-side colon cancer (RCC), left-side colon cancer (LCC), and rectal cancer (RECC) among Chinese patients with CRC. We evaluated 4,426 patients with stage I–III CRC between January 2008 and July 2014from Fudan University Shanghai Cancer Center. All patients were grouped by the locations of tumors (RCC, LCC, and RECC). Patients with RCC were more likely to be women and older, have poorly differentiated tumors, microsatellite repair deficiency (dMMR), negative p53 expression, and the mucinous subtype. Unadjusted Kaplan-Meier survival curves revealed survival in RCC than in LCC and RECC. However, there were no significant differences in OS and DFS between LCC and RECC. The same results were observed for each disease stage. Unadjusted models revealed an increased risk of mortality, recurrence, or metastasis for RCC (OS: HR, 1.68, *P*=0.0002 and DFS: HR, 1.24, *P*=0.032), compared to LCC (all stages), and a similar result was observed for stage III patients (OS: HR, 1.79, *P*<0.0001 and DFS: HR, 1.33, *P*=0.021). However, adjusted Cox proportional hazard regression models revealed no significant differences in survival between the three tumor locations. Tumor location was not an independent prognostic factor among Chinese patients with stage I-III CRC. But RCCs had a worse prognosis in the dMMR subgroup. The related mechanism remains to be investigated.

## INTRODUCTION

Colorectal cancer (CRC) is one of the most commonly diagnosed cancers worldwide [1]. During the past decades, interest has increased on the distribution of CRC throughout the different segments of the colon and rectum. Previous studies have evaluated various CRC characteristics, including its epidemiology, pathological features, and molecular pathogeneses, based on the primary tumor location [2–5]. In 1990, Bufill originally proposed that right-side colon cancers (RCCs) and left-side colon cancers (LCCs) might arise via distinct biological pathways. Furthermore, the embryological developments of the two colon segments are different, which may explain the different molecular and biological tumor patterns [6].

Several studies have demonstrated that RCCs are typically bulky exophytic polypoid lesions that project into the lumen and cause significant anemia. In contrast, LCCs are infiltrating and constricting lesions that encircle the lumen and often lead to obstruction [7], including poor differentiation, the mucinous type, a larger size, higher TNM stage, vessel invasion, and an expanding tumor border. Moreover, the microsatellite instability (MSI) that is associated CRC tends to occur in the proximal part of the colon, and chromosomal instability (CIN) tends to occur in the distal part of the colon [8].

Recent studies have highlighted that RCCs exhibit a shorter survival, compared to LCCs [9]. Venook et al. [10] advocated that in KRAS wildtype metastatic CRC (mCRC), patients with left-sided primary tumor have superior overall survival (OS) and progression free survival (PFS) compared with right-sided primary tumor patients. Moreover, OS and PFS were prolonged in left-sided mCRC using Cetuximab and right-sided mCRC using Bevacizumab, but were poorer in right-sided mCRC using Cetuximab. However, other researchers have claimed that there is no statistically significant survival difference by tumor location among patients with CRC. Rectal cancer (RECC) is considered a separate entity in CRC, as RECC has different pathological and molecular features, compared to RCC and LCC [3, 11, 12]. Few studies have compared the survival rates between RCC, LCC, and RECC. In the present study, we compared the pathological characteristics, molecular features, and prognoses between RCC, LCC, and RECC, in order to investigate whether patients with CRC exhibit different survivals and characteristics by tumor location.

## RESULTS

### Patient characteristics

Among the 4,426 patients, 58.29% were men and 41.71% were women. The cases were most commonly diagnosed between the ages of 51 years and 75 years (67.65%), with21.08% of the tumors being poorly differentiated and 72.19% being moderately differentiated. The most common pathological type was ulcerative (54.47%), followed by protruded (31.27%), complex (8.31%), and infiltrating types (2.21%); 86.47% of patients had a non-mucinous tumor. The majority of cases were diagnosed with stage III CRC (47.42%), which was followed by stage II CRC (32.92%). The dMMR subset accounted for 22.73% of patients, and 64.37% of the cases exhibited positive p53 expression.

Among all cases, 20.9% (n = 927) of the patients had RCC, 21.8% (n=966) had LCC, and 57.2% (n=2,533) had RECC. There were significant differences between the tumor locations regarding their sex, age distribution, stage, grade, dMMR status, histological subtype, and pathological type (all, *P*<0.05). Compared to LCC and RECC, RCC cases were more likely to be women (46.60% vs. 40.37% and 40.43%) and elderly (age of >65 years: 35.81% vs. 31.36% and 26.17%, respectively). Cases of RCC exhibited a lower frequency of stage I tumors (11.54% vs.15.01% and 24.40%, respectively) and higher frequencies of poorly differentiated tumors (28.69% vs.18.63% and 19.23%, respectively), dMMR (31.07%vs.21.84% and 20.02%, respectively), negative p53 expression (31.18% vs.26.60% and 27.67%, respectively), and the mucinous subtype (21.68% vs.11.59% and 10.58%, respectively) Table 1. The patients' characteristics by stage and location are listed in Table 2.

**Table 1:** Demographic and Clinicopathological Features of Patients with Colorectal Cancer

Features	All	RCC	LCC	RECC	*P*-value
	(N=4426)	(n=927)	(n=966)	(n=2533)	
Gender					
Male	58.29	495(53.4%)	576(59.6%)	1509(59.6%)	0.0031
Female	41.71	432(46.6%)	390(40.4%)	1024(40.4%)	
Age (y)					
<50	23.5	196(21.1%)	226(23.4%)	618(24.4%)	<0.0001
51-65	47.18	399(43.0%)	437(45.3%)	1252(49.4%)	
66-75	20.47	224(24.2%)	208(21.5%)	474(18.7%)	
>75	8.86	108(11.7%)	95(9.8%)	189(7.5%)	
Grade					
1	21.08	266(28.7%)	180(18.6%)	487(19.2%)	<0.0001
2	72.19	579(62.5%)	733(75.9%)	1883(74.3%)	
3	2.06	12(1.3%)	27(2.8%)	52(2.1%)	
Unknown	4.68	70(7.5%)	26(2.7%)	111(4.4%)	
Histologic type					
Non-mucinous	86.47	724(78.1%)	848(87.8%)	2255(89.0%)	<0.0001
Mucinous	13.13	201(21.7%)	112(11.6%)	268(10.6%)	
Unknown	0.41	2(0.2%)	6(0.6%)	10(0.4%)	
Pathological type					
Complex	8.31	76(8.2%)	85(8.8%)	207(8.2%)	0.0178
Infiltrating	2.21	26(2.8%)	15(1.6%)	57(2.2%)	
Ulcerative	54.47	507(54.7%)	556(57.6%)	1348(53.2%)	
Protruded	31.27	270(29.1%)	278(28.8%)	836(33.0%)	
Unknown	3.73	48(5.2%)	32(3.4%)	85(3.4%)	
T stage					
1	5.99	28(3.0%)	53(5.5%)	184(7.3%)	<0.0001
2	18.93	94(10.1%)	127(13.1%)	617(24.4%)	
3	48.26	236(25.5%)	196(20.3%)	1704(67.3%)	
4	26.28	562(60.6%)	586(60.7%)	15(0.5%)	
Unknown	0.54	7(0.8%)	4(0.4%)	13(0.5%)	
N stage					
0	52.58	486(52.4%)	520(53.8%)	1321(52.1%)	0.0647
1	26.21	257(27.7%)	265(27.4%)	638(25.2%)	
2	21.22	184(19.9%)	181(18.8%)	574(22.7%)	
Stage					
I	19.66	107(11.5%)	145(15.0%)	618(24.4%)	<0.0001
II	32.92	379(40.9%)	375(38.8%)	703(27.8%)	
III	47.42	441(47.6%)	446(46.2%)	1212(47.8%)	
MMR					
dMMR	22.73	288(31.0%)	211(21.8%)	507(20.0%)	<0.0001
pMMR	64.89	511(55.1%)	648(67.1%)	1713(67.6%)	
Unknown	12.38	128(13.9%)	107(11.1%)	313(12.4%)	
p53*					
0	28.17	289(31.2%)	257(26.6%)	701(27.7%)	0.0549
1	64.37	562(60.6%)	647(67.0%)	1640(64.7%)	
Unknown	7.46	76(8.2%)	62(6.4%)	192(7.6%)	
CEA	Median	3.48	3.34	3	<0.0001
	Range	(0-1041.0)	(0.2-1000.0)	(0.2-1036.0)	

Abbreviations: RCC-right-side colon cancer, LCC-left-side colon cancer, RECC-rectal cancer, MMR-microsatellite repair deficiency, dMMR-MMR deficiency, pMMR-MMR proficiency, CEA-carcino-embryonic antigen

*IHC of p53 was detected mutation protein.

**Table 2:** Demographic and Clinicopathological Features of Patients Stratified by Stages

Patients (%)												
		Stage I (n=870)				Stage II (n=1457)				Stage III (n=2099)		
	RCC	LCC	RECC	P-value	RCC	LCC	RECC	P-value	RCC	LCC	RECC	P-value
	(n=107)	(n=145)	(n=618)		(n=379)	(n=375)	(n=703)		(n=441)	(n=446)	(n=1212)	
Gender												
Male	56.1	51.7	52.8	0.772	53.3	62.4	64.9	<.001	52.8	59.9	60.0	0.026
Female	43.9	48.3	47.3		46.7	37.6	35.1		47.2	40.1	40.0	
Age												
<50	20.6	16.6	19.4	0.051	20.8	24.53	20.5	0.112	21.5	24.7	29.2	<.001
51-65	43	46.2	53.6		42	41.6	48.1		44	48	48.1	
66-75	25.2	30.3	19.1		23	21.3	21.8		25	18.8	16.8	
>75	11.2	6.9	7.9		14.2	12.5	9.7		9.5	8.5	5.9	
Grade												
1	14	7.6	9.6	0.0017	27.2	15.7	13.1	<.001	33.6	24.7	27.7	<0.001
2	72	76.6	79.5		66	80.3	82.5		57.1	72	67	
3	2.8	12.4	6.2		1.1	1.6	1.1		1.1	0.7	0.5	
Unknown	11.2	3.5	4.8		5.8	2.4	3.3		8.2	2.7	4.8	
Histological types												
Non-mucinous	80.4	97.2	91.9	<.001	77.8	89.1	89.2	<.001	77.78	83.63	87.46	<.001
Mucinous	19.6	2.8	7.3		21.9	9.9	10.8		22	15.92	12.13	
Unknown	0	0	0.8		0.3	1.1	0		0.23	0.45	0.41	
Type												
Complex	5.6	2.8	4.7	0.013	9	10.4	9.1	0.299	8.16	9.42	9.41	0.176
Infiltrating	1.9	0	0.97		2.4	0.8	2.8		3.4	2.69	2.56	
Ulcerative	16.8	23.5	29.6		56.4	60	60.5		62.4	66.6	61.1	
Protruded	63.6	64.1	59.6		28.8	26. 7	25.7		21.1	19.1	23.7	
Unknown	12.2	9.7	5.2		3.4	2.1	1.9		4.99	2.24	3.3	
MMR												
dMMR	28	24.8	17.6	0.0002	34.8	25.1	21.9	<.001	28.6	18.2	20.1	<.001
pMMR	41.1	55.9	63.8		54.4	66.7	69.7		59.2	71.1	68.4	
Unknown	30.8	19.3	18.6		10.8	8.3	8.4		12.2	10.8	11.5	
p53*												
0	33.6	31	26.2	0.174	31.4	24.5	28.9	0.182	30.4	26.9	27.7	0.415
1	47.7	55.9	59.7		62.8	71.2	66.4		61.9	67	66.3	
Unknown	18.7	13.1	14.1		5.8	4.3	4.7		7.7	6.1	5.9	

Abbreviations: RCC-right-side colon cancer, LCC-left-side colon cancer, RECC-rectal cancer, MMR-microsatellite repair deficiency, dMMR-MMR deficiency, pMMR-MMR proficiency

*IHC of p53 was detected mutation protein.

### Survival analyses by tumor stage and location

**Figure 1:**
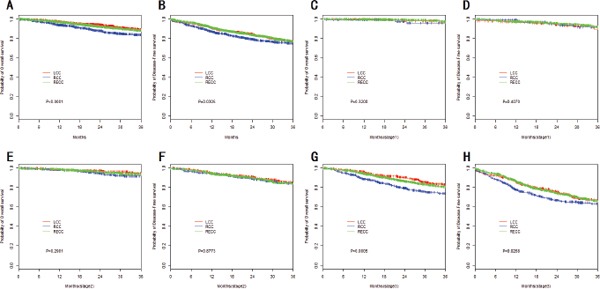
Kaplan–Meier survival analyses by tumor stage and location **(A)** OS of all patients; **(B)** DFS of all patients; **(C)** OS of stage I patients; **(D)** DFS of stage I patients; **(E)** OS of stage II patients; **(F)** DFS of stage II patients; **(G)** OS of stage III patients; **(H)** DFS of stage III patients.

For all stages, the unadjusted Kaplan-Meier survival curves revealed significantly better OS among LCC cases, compared to RCC cases. However, there was no significant difference between the OS of the RECC and LCC cases (all stages) (Figure 1A). Similar findings were observed in the stage subanalyses (Figure 1C, 1E, 1G). Unadjusted survival models considering tumor location variable revealed an increased risk of mortality for RCC, compared to LCC, for all stages (HR, 1.68;95% CI, 1.28–2.21;*P*=0.0002). Patients with stage III RCC also had a higher risk of mortality (HR, 1.79; 95% CI, 1.30–2.46; *P*<0.0001), although no significant location-specific differences in OS were observed for patients with stage I and II disease (Table 3). Additional analyses were performed using adjusted Cox proportional hazard regression models, and no significant differences were observed for LCC, RECC, and RCC (all stages) after controlling for age, sex, CEA, histological subtype, tumor grade, pathological type, p53 expression, and MSI (Table 3).

**Table 3:** Adjusted Hrs And 95%Cis For Mortality By Stage

Analysis Type	All Stages Combined (N=4426)			Stage I (n=870)			Stage II (n=1457)			Stage III (n=2099)		
	HR	95%CI	P	HR	95%CI	P	HR	95%CI	P	HR	95%CI	P
Unadjusted												
Left colon	1.00			1.00			1.00			1.00		
Rectum	1.12	0.88-1.44	0.353	0.53	0.18-1.54	0.240	1.14	0.66-1.97	0.635	1.21	0.91-1.62	0.187
Right colon	1.68	1.28-2.21	0.0002	1.08	0.29-4.04	0.906	1.53	0.86-2.74	0.148	1.79	1.30-2.46	0.000
Adjused*												
Left colon	1.00			1.00			1.00			1.00		
Rectum	1.02	0.76-1.36	0.900	0.54	0.11-2.57	0.439	1.12	0.61-2.07	0.713	1.07	0.76-1.51	0.701
Right colon	1.24	0.88-1.73	0.215	1.88	0.17-22.3	0.611	1.27	0.63-2.56	0.506	1.34	0.90-1.99	0.145

Abbreviations: HR, hazard ratio

*Cox regression model controlling for age; gender; CEA; histologic subtypes; tumor grade; tumor pathological type and P53 Microsatellite instability.

Univariate Kaplan-Meier DFS curves also revealed that LCC exhibited a significantly longer DFS than RCC (all stages). However, no significant difference was found between RECC and LCC (all stages) (Figure 1B). The stage subanalyses reached similar findings (Figure 1D, 1F, 1H). Unadjusted models revealed an increased risk of recurrence or metastasis for RCC, compared to LCC, for all stages (HR, 1.24;95% CI, 1.02–1.52;*P*=0.032). Patients with stage III RCC also had a higher risk of mortality (HR, 1.33;95% CI, 1.04–1.69;*P*=0.021), but for stage I and II disease we did not find such difference (Table 4). After adjusting for various confounders, no significant difference in DFS was observed for LCC, RECC, and RCC (all stages) (Table 4).

**Table 4:** Adjusted Hrs And 95%Cis For Recurrence Or Metastasis By Stage

Analysis Type	All Stages Combined (N=4426)			Stage I (n=870)			Stage II (n=1457)			Stage III (n=2099)		
	HR	95%CI	P	HR	95%CI	P	HR	95%CI	P	HR	95%CI	P
Unadjusted												
Left colon	1.00			1.00			1.00			1.00		
Rectum	0.99	0.84-1.18	0.964	0.66	0.35-1.25	0.200	1.03	0.72-1.48	0.854	1.04	0.85-1.29	0.691
Right colon	1.24	1.02-1.52	0.032	0.63	0.24-1.67	0.355	1.11	0.74-1.66	0.621	1.33	1.04-1.69	0.021
Adjused*												
Left colon	1.00			1.00			1.00			1.00		
Rectum	0.98	0.80-1.21	0.865	0.55	0.23-1.35	0.195	1.12	0.74-1.71	0.595	1.02	0.80-1.31	0.860
Right colon	1.10	0.86-1.40	0.470	0.20	0.02-1.89	0.162	1.06	0.64-1.73	0.831	1.19	0.89-1.60	0.240

HR, hazard ratio

*Cox regression model controlling for age; gender; CEA; histologic subtypes; tumor grade; tumor pathological type and P53 Microsatellite instability.

### Subset analyses

**Figure 2:**
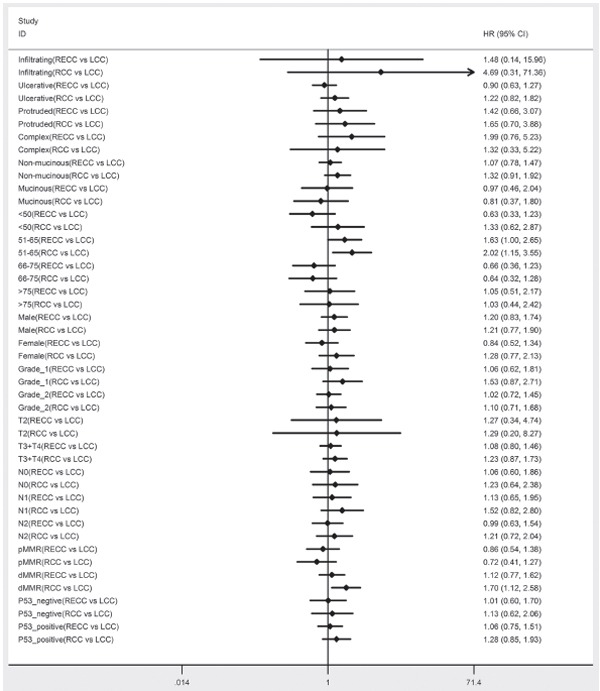
Adjusted hazard ratio with 95% CIs for OS comparing RECC to LCC and comparing RCC to LCC in the different cohort

**Figure 3:**
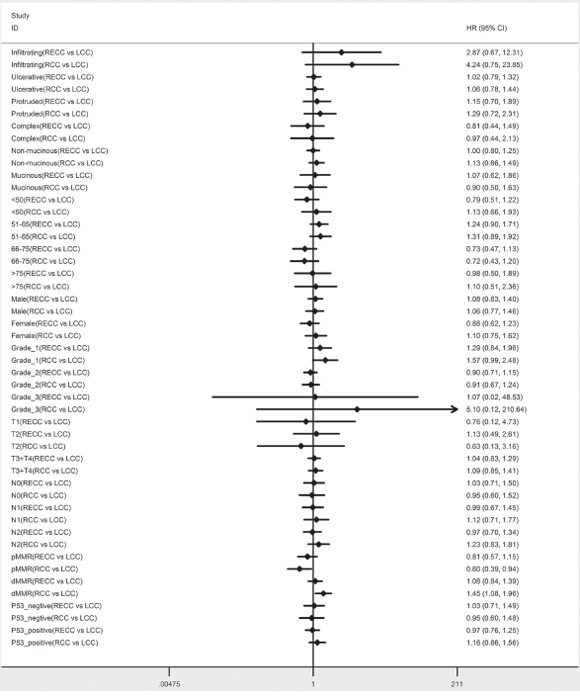
Adjusted hazard ratio with 95% CIs for DFS comparing RECC to LCC and comparing RCC to LCC in the different cohort

The adjusted Cox proportional hazard regression analyses were performed in various subsets. Most subsets exhibited no significant difference for RCC and RECC, compared to LCC (the reference group). In the subset analyses of OS, a significant difference was observed between LCC and RCC for patients who were 51–65 years old (HR, 2.02; 95%CI, 1.15–3.55;*P*=0.014) and dMMR (HR. 1.70; 95%CI, 1.12–2.58;*P*= 0.013) (Figure 2). In the subset analyses of DFS, a significant difference was observed between LCC and RCC for patients who wered MMR (HR, 1.45; 95%CI, 1.08–1.96;*P*= 0.013) and pMMR (HR, 0.60; 95%CI, 0.39–0.94;*P*= 0.026) (Figure 3).

## DISCUSSION

In the present study, we compared the clinicopathological and molecular features of three CRC tumor locations. Elderly patients and women were more likely to have RCC. Interestingly, the symptoms of RCCs tend to appear later than those of the other two sites, and colonoscopies may not be completed due to pain or technical problems. These factors may explain why our elderly patients were more likely to have RCCs. Sex-related hormones may also affect the relative frequency of RCCs among women [13–15]. Our stage-based analysis revealed that RCCs consisted of a greater proportion of stage II tumors, high T stage cancers, and poorly differentiated carcinomas and tumors with mucinous or signet-ring cell components (vs. the other locations). These results are consistent with the findings of previous studies [16]. We also found that dMMR tumors were more common among RCCs, which were also more likely to be negative for p53 expression. These findings may be attributed to the different biological pathogeneses at the various locations for CRC, which may influence the development of the tumor. Furthermore, we compared the clinicopathological and molecular features by location and stage. Most findings of the subgroup analyses were statistically significant (with similar trends), although no significant differences were observed for sex in stage I, age in stages I and II, and p53 expression in all three stages.

In previous decades, research has concentrated on the relationships between CRC location and patient survival. However, controversy remains regarding whether RCCs have a worse prognosis. Most previous studies have addressed the difference in mortalities between RCCs and LCCs, and have excluded RECCs or combined them with LCCs [17, 18]. In our study, all three locations were evaluate as distinct groups, and our univariate analysis revealed shorter OS and DFS in RCCs (vs. LCCs and RECCs, *P*<0.001), although RECCs and LCCs exhibited similar OS and DFS. However, when the patients were stratified by stage, we did not observe any significant differences in OS and DFS between LCCs, RCCs, and RECCs for stage I and stage II cancer. This may be partially related to the fact that patients with stage I and stage II CRC generally have good prognosis. MSI was more common in stage II CRC, and MSI occurred most frequently in RCC. MSI-associated CRC cases have a better prognosis, which may also partially explain our findings. Furthermore, after controlling for age, sex, CEA, histological subtype, tumor grade, pathological type, p53 expression, and MMR, we found no significant difference in the OS and DFS between the three CRC locations. That finding appears to indicate that age, differentiation, stage, or CEA level may have a stronger prognostic value for CRC, compared to tumor location.

There are few studies that have compared survival by the three primary locations in CRC, and their findings are consistent with those of the present study. Powell et al. [19] evaluated 411 patients who had undergone surgery for stage I–III CRC, and the authors reported no significant differences in survival by location in their univariate analysis. However, Suttie et al. [20] found that RCCs had a worse prognosis than LCCs and RECCs in their univariate analysis. Nevertheless, given their relatively small sample sizes, these studies may not have provided sufficient power for the analyses. In contrast, we used a large sample size. Although our findings are consistent with those of several similar studies, important differences are also present. For example, Weiss et al. [21] evaluated 53,801 patients with stage I–III primary adenocarcinoma of the colon using the Surveillance, Epidemiology, and End Results (SEER) database. Similar to our findings, their unadjusted survival analysis revealed that RCC had a worse survival than LCC (all stages). However, among their stage II subgroup, RCC exhibited a lower mortality, and no significant difference was observed in the adjusted survival rates by the tumor locations (all stages) (HR, 1.01; 95% CI, 0.98–1.04;*P* = 0.598) or for stage I cancers (HR, 0.95; 95% CI, 0.88–1.03; *P* = 0.211). Stage II RCC had a significantly better prognosis (HR, 0.92; 95% CI, 0.87–0.97;*P* = 0.001), and stage III RCCs exhibited a significantly worse prognosis (HR, 1.12; 95% CI, 1.06–1.18; *P* = 0.001). Based on those findings, Weiss et al. concluded that these differences were most likely related to tumor biology, and especially to MSI. In our study, we found no significant difference in the survival rates for all locations and stages. One possible explanation is that our findings were limited by the sample size. Another study of SEER data by Meguid et al. [22] demonstrated that, after the multivariate Cox proportional hazard regression analyses, RCC was associated with a statistically significant increase in the risk of mortality, compared to LCC (all stages). Moreover, they reported a better survival outcome for RCC, compared to LCC, in stage II CRC, which is consistent with Weiss et al.'s findings. However, the cause of that relationship remains unclear and requires further study. Benedix et al. [23] evaluated 17,641 patients with CRC and found that RCC had a higher risk of mortality, compared to LCC, in their proportional hazard model. However, the effects of tumor location on survival were smaller than those of other factors, including age and tumor stage. Furthermore, all three studies contain significant limitations. For example, although each study included a relatively large sample size, multicenter patient populations invariably receive different medical treatments. In addition, only 77.9% of patients completed the follow-up in Benedix et al.'s study, which may have influenced their survival outcomes. In contrast, all patients in the present study had complete follow-up data, which ensures a relatively high accuracy for the survival analyses. Therefore, the different sample sizes and enrollment criteria may explain the inter-study differences, and race may also influence the studies' results. Recently, a review and meta-analysis by Yahagi et al. [24] advocated that RCC have a significantly worse prognosis than those with LCC in terms of OS. But through their subgroup analysis, it showed that significant difference in prognosis between RCC and LCC was identified only in Western countries, while it was inconsistent in Eastern countries. Accordingly, association between tumor location and prognosis may not be as strong as we had assumed in Chinese CRC patients.

In the study by Weiss et al., the authors hypothesized that the inconsistent relationship between mortality and tumor location by stage was likely related to MSI. This may be partially correct, given the fact that patients with MSI-high status have a better prognosis, and that MSI-high tumors are predominantly observed in stage II CRC. However, those authors did not perform any subgroup analyses. In the present study, we performed an MMR subgroup analysis and identified that RCC had a shorter OS in patients who were 51–65 years old and dMMR. Similarly, RCC had a worse prognosis for DFS in the dMMR subset and a better prognosis in the pMMR subset. Tougeron et al. [25] retrospectively analyzed 433 patients with stage II or III dMMR colorectal cancer, they found there is no significant difference in DFS. Another research held by Sargent showed that MMR status alone was not significantly associated with the disease recurrence time [26]. Sinicrope et al. [27] proved that dMMR showed improved outcome compared with pMMR for time to recurrence, DFS and OS in stage II and stage III disease. But it was no statistically different only in stage II disease. And in stage III, dMMR patients had a shorter time to recurrence (p<0.05). However, most of the published researches did not analyze the relationship of MMR status and tumor location with colorectal cancer survival.

This present study compared the survival rates between RCC, LCC, and RECC of stage I-III patients in a large Chinese cohort. There were no difference in the survival of Chinese CRC patients with different tumor locations, which indicates ethnic effector could contribute to the issue. However, limitations also exist, because in this was a single-center experience bias was inevitable. Multicenter data analyses should be explored in the future to test our results in Chinese CRC patients. Besides, stage IV patients was not included in this research. Further study should be conducted to test whether the tumor location would affect the treatment or survival in these patients.

In conclusion, tumor location was not an independent prognostic factor among Chinese patients with stage I-III CRC. However, in the dMMR subgroup, patients with RCC have a worse prognosis. The mechanism for this association remains to be investigated.

## MATERIALS AND METHODS

### Patients

We retrospectively reviewed 4,426 CRC cases that were treated at Fudan University Shanghai Cancer Center between January 2008 and July 2014. The inclusion criteria were (1) patients with pathologically confirmed CRC, (2) patients who had undergone curative surgical resection, and (3) patients with stage I–III CRC. We excluded patients who had (1) undergone preoperative chemotherapy or radiotherapy, (2) two or more primary tumors, or (3) tumors with an unknown location. Disease staging was performed according to the fifth edition of the American Joint Committee on Cancer's TNM classification. The patients' demographic and clinicopathological characteristics were collected from a medical data platform by trained staff, who used standardized data collection and quality-control procedures.

The following parameters were analyzed for all patients: age at diagnosis, sex, tumor location, tumor differentiation, TNM stage, histological subtype, pathological type, carcinoembryonic antigen (CEA) level, p53expression, and microsatellite instability (MSI). Patients who were negative for the expression of human mutL homolog 1 (hMLH1), human mutS homolog 2 (hMSH2), and/or human mutS homolog 6 (hMSH6) were defined as having mismatch repair deficiency (dMMR), and all other patients were defined as having mismatch repair proficiency (pMMR). The primary explanatory variable was tumor location (LCC, RCC, or RECC). LCCs included tumors in the splenic flexure, descending colon, and sigmoid colon. RCCs included tumors in the cecum, ascending colon, hepatic flexure, and transverse colon.

### Follow-up

Follow-up data were collected from the Fudan University Shanghai Cancer Center's follow-up platform, or from the tumor registry database of the Shanghai Municipal Center for Disease Control & Prevention if the patient was lost to follow-up. Overall survival (OS) was defined as the time from initial surgical resection until death due to any cause. Disease-free survival (DFS) was defined as the time from the initial surgical resection to recurrence or metastasis of CRC. Patients who were alive at the last follow-up were censored. The median duration of follow-up for all participants was 28.3 months (range: 0.1–85.7 months).

### Statistics analysis

Data for all categorical variables were summarized as frequencies, and data for all continuous variables were presented as median and range. The chi-square test was used to compare differences in the distributions and proportions of the demographic and clinicopathological variables by tumor location. Survival curves were calculated using the Kaplan-Meier method, and survival functions were compared using the log-rank test. Unadjusted and adjusted Cox proportional hazards regression analyses were used to estimate the association between tumor location and outcomes (OS and DFS), and to obtain the corresponding hazard ratios (HRs) and 95% CIs for the different predictors. The adjusted Cox regression models included age, sex, CEA, histological subtype, tumor grade, pathological type, p53 expression, and MSI. The effects of primary tumor location on OS and DFS were also assessed in subset analyses that were stratified by several baseline variables. Differences were considered statistically significant for a *P*-value of <0.05. All statistical analyses were performed using SAS software (version 9.4; SAS Institute Inc., Cary, NC).

## SUPPLEMENTARY TABLES


