# Supplementary material for: Is there a prognostic value of tumor location among Chinese patients with colorectal cancer?
Source: Oncotarget. 2017 Mar 17;8(24):38682–92. doi: 10.18632/oncotarget.16305 (PMC5503563; doi:10.18632/oncotarget.16305)
Supplement: Supplementary file 1 [file oncotarget-08-38682-s001.pdf]

Is there a prognostic value of tumor location among Chinese patients with colorectal cancer?

SUPPLEMENTARY TABLES

Supplementary Table 1: Interactions (age, gender, dMMR and p53 with tumor location) associated with mortality using Cox proportional hazards regression analyses

Interaction	Cox analysis	
	HR(95% CI)	*P*
Age*Tumor location	1.04(1.00 ~1.07)	0.030
Gender*Tumor location	0.99(0.93~1.06)	0.817
dMMR*Tumor location	0.90(0.83~0.97)	0.007
P53*Tumor location	0.93 (0.87~1.00)	0.081

Supplementary Table 2: Interactions (age, gender, dMMR and p53 with tumor location) associated with recurrence or metastasis using Cox proportional hazards regression analyses

Interaction	Cox analysis	
	HR(95% CI)	*P*
Age*Tumor location	1.00(0.97 ~1.02)	0.780
Gender*Tumor location	0.03(0.973~1.08)	0.353
dMMR*Tumor location	0.94(0.89~1.00)	0.038
P53*Tumor location	0.96 (0.91~1.01)	0.168
